# Primary Peritoneal Carcinoma: A Rare Malignancy Presenting a Diagnostic Challenge

**DOI:** 10.7759/cureus.25082

**Published:** 2022-05-17

**Authors:** Mariam Shabbir, Sonu Sahni, Meena Ahluwalia, Raji Ayinla

**Affiliations:** 1 Internal Medicine, NYC Health + Hospitals/Harlem - Columbia University, New York, USA; 2 Pulmonary and Critical Care Medicine, NYC Health + Hospitals/Harlem - Columbia University, New York, USA; 3 Primary Care, Touro College of Osteopathic Medicine, New York, USA; 4 Hematology and Oncology, NYC Health + Hospitals/Harlem - Columbia University, New York, USA

**Keywords:** mullerian tumor, immuno-histochemical, diagnostic delay, epithelial ovarian cancer, primary peritoneal carcinoma

## Abstract

With the advancement in technologies and the development of a vast variety of tests, diagnosing diseases has become relatively easy. However, certain diseases are challenging to diagnose due to their similarities with other disease processes. Primary peritoneal carcinoma (PPC) is one of the infrequent tumors that has a resemblance to an ovarian tumor, often making it hard to diagnose. The symptoms are non-specific, and by the time primary peritoneal cancer is diagnosed, the patient is usually at an advanced stage. Although diagnosis might be suspected based on presenting symptoms, it is rarely confirmed with symptomatology alone, requiring additional tumor markers or radiological studies. Sometimes it is diagnosed after surgical removal of the lesion. Several similarities have been described between PPC and ovarian cancer, with some studies explaining the differences as well. We highlight the importance of careful interpretation of imaging studies for the timely diagnosis of PPC. However, several factors can interfere with the analysis of test results leading to delays in diagnosis and management. Interpretation of imaging becomes difficult, especially in patients with significant ascites.

## Introduction

Primary peritoneal carcinoma (PPC) is a rare and aggressive tumor that ordinates from the lining of the peritoneum. The most common presenting symptoms include abdominal distension, abdominal pain, a sense of fullness, nausea, emesis, constipation, increased urinary frequency, dyspnea, fatigue, and weight loss. Diagnosis and treatment of PPC are time-sensitive due to the rapidly progressive nature of the disease. Identification of PPC may be difficult due to its resemblance to ovarian cancer. For an expeditious and definitive diagnosis, extensive studies are required, including specific tumor markers, imaging in the form of computed tomography (CT), ascitic fluid analysis including cytology, and immunohistochemistry. The Gynecologic Oncology Group has established the following diagnostic criteria for PPC: (1) Both ovaries are normal in size or enlarged due to a benign process; (2) the involvement in extraovarian sites is greater than the involvement on the surface of either ovary; (3) microscopically, the ovaries are not involved by the tumor or exhibit only serosal or cortical invasion with dimensions smaller than 5.0 × 5.0 mm; (4) the histopathological characteristics of the tumor are predominantly of the serous type [1]. Based on the criteria, once a diagnosis of PPC is confirmed, surgical cytoreduction and chemotherapy are the main therapeutic options. Here, we present the case of a 58-year-old woman who was initially thought to have ovarian cancer; however, an extensive workup showed evidence of PPC of Müllerian origin. Through this case, we highlight the importance of careful analysis and interpretation of test results in establishing a definitive diagnosis, especially in the presence of confounding factors.

## Case presentation

A 58-year-old African American woman presented to the emergency department of our institution with a chief complaint of diffuse abdominal pain with abdominal distension associated with constipation of one month’s duration and unintentional weight loss of 10 pounds over the course of two months. Her medical history was significant for chronic hepatitis B infection, human immunodeficiency virus (HIV) (absolute CD4 count 398/µL), type II diabetes mellitus, hypertension, and asthma. Vital signs on admission were as follows: heart rate of 128 beats/minute, blood pressure of 125/75 mmHg, temperature of 97.2°F, respiratory rate of 18 breaths/minute, and oxygen saturation of 92% on room air. Initial laboratory investigations were remarkable for acute kidney injury with a creatinine of 2.1 mg/dL (baseline 1.0 mg/dL) and blood urea nitrogen elevated at 33 mg/dL. Human chorionic gonadotropin in serum was elevated at 7 mIU/mL (normal range ≤ 5 mIU/mL). Laboratory investigations are presented in Table 1.

**Table 1:** Laboratory investigations.

Component	Reference ranges	Patient values
Serum		
Lactate dehydrogenase	135–214 U/L	261 U/L
Complete blood count		
Hemoglobin	12.0–16.0 g/dL	13.9 g/dL
Hematocrit	37.0–47.0%	45.4%
Mean corpuscular volume	80.0–99.0 fL	98.7 fL
White blood cell count	4.80–10.80 × 10^3^/µL	5.92 × 10^3^/µL
Platelet count	150–450 × 10^3^/µL	286 × 10^3^/µL
Basic metabolic panel		
Sodium	136–145 mmol/L	138 mmol/L
Potassium	3.5–5.1 mmol/L	5.5 mmol/L
Chloride	98–107 mmol/L	104 mmol/L
Bicarbonate	22–29 mmol/L	18 mmol/L
Blood urea nitrogen	7–18 mg/dL	33 mg/dL
Creatinine	0.5–0.9 mg/dL	2.1 mg/dL
Glucose	74–109 mg/dL	80 mg/dL
Calcium	8.5–10.1 mg/dL	9.5 mg/dL
Anion gap	6–18 mEq/L	16 mEq/L
Estimated glomerular filtration rate	≥60 mL/minute/1.73 m^2^	25 mL/minute/1.73 m^2^
Hepatic function panel		
Albumin	3.97–4.94 g/dL	4.0 g/dL
Total protein	6.4–8.3 g/dL	7.8 g/dL
Total bilirubin	≤1.2 mg/dL	0.8 mg/dL
Direct bilirubin	0.0–0.3 mg/dL	<0.2 mg/dL
Alkaline phosphatase	35–104 U/L	154 U/L
Aspartate transaminase	≤33 U/L	14 U/L
Alanine transaminase	≤32 U/L	32 U/L
Tumor markers		
Cancer antigen 125	≤38 U/mL	214 U/mL
Cancer antigen 19-9	≤35 U/mL	53 U/mL
Carcinoembryonic antigen	0–3.8 ng/mL	3.5 ng/mL
Ascitic fluid studies		
Glucose	Reference range has not been established due to variability in body fluids	18 mg/dL
Lactate dehydrogenase	Reference range has not been established due to variability in body fluids	855 IU/L
Protein	Reference range has not been established due to variability in body fluids	3.8 g/dL

**Figure 1:**
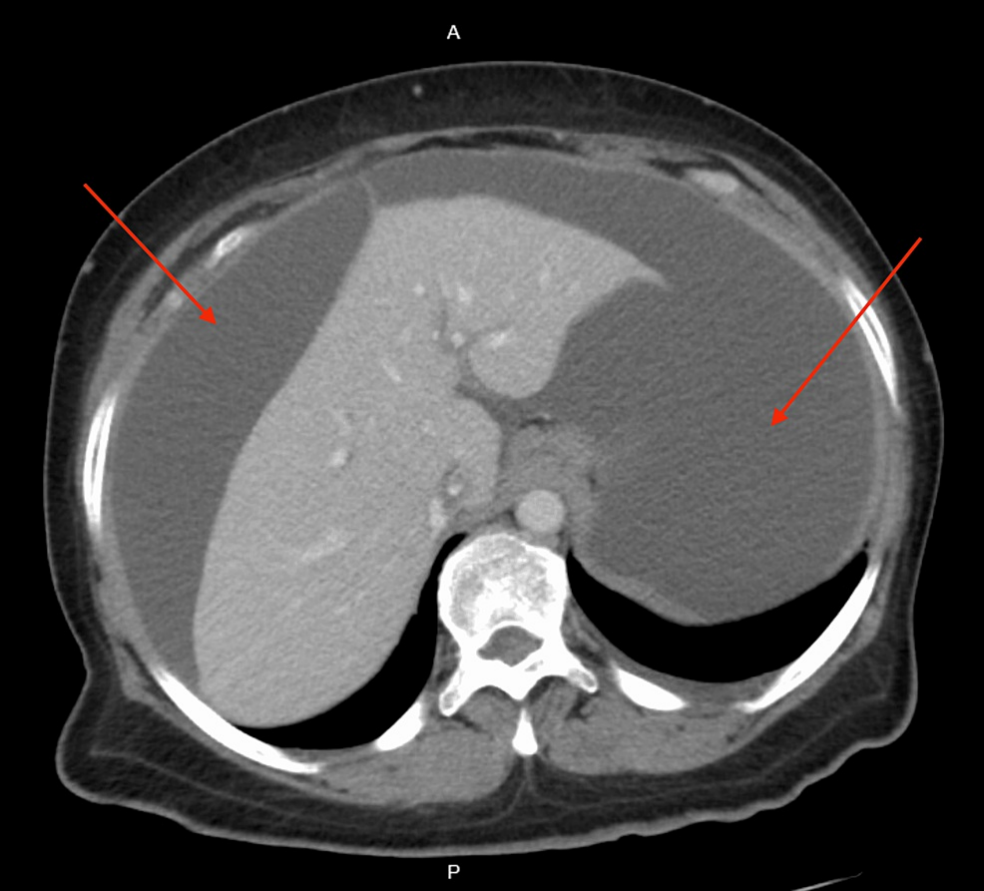
Computed tomography of the abdomen with intravenous contrast showing large volume ascites.

**Figure 2:**
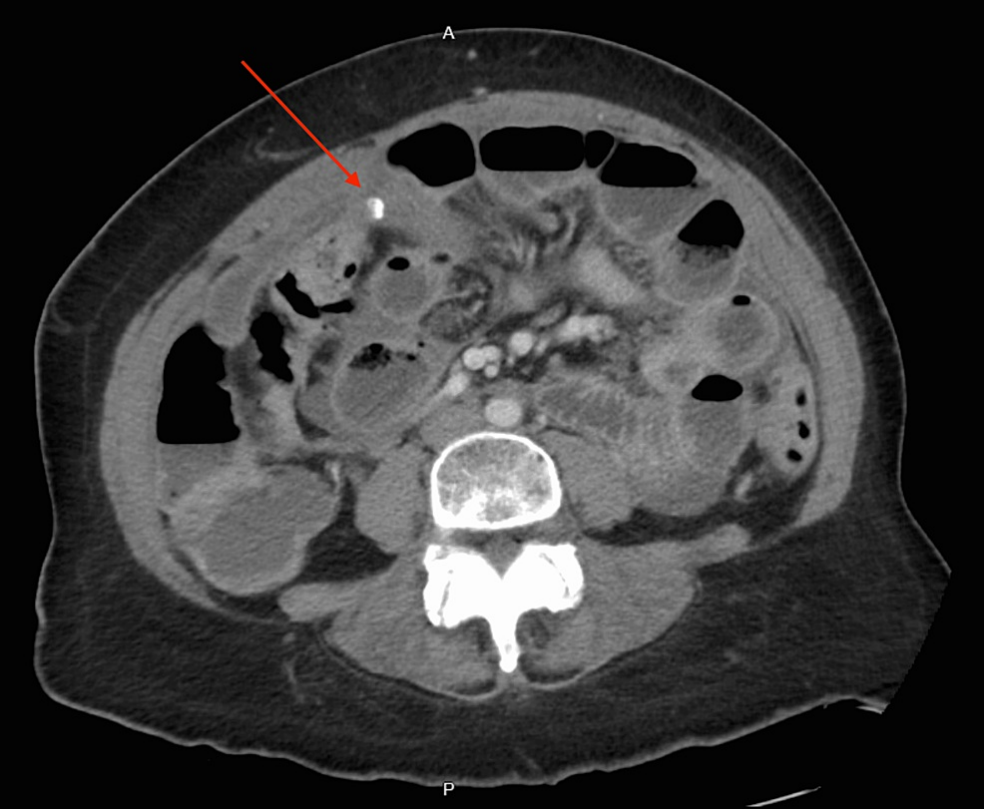
Computed tomography of the abdomen with intravenous contrast showing a soft tissue density with an 8 mm focal area of calcification with distortion of the omentum.

**Figure 3:**
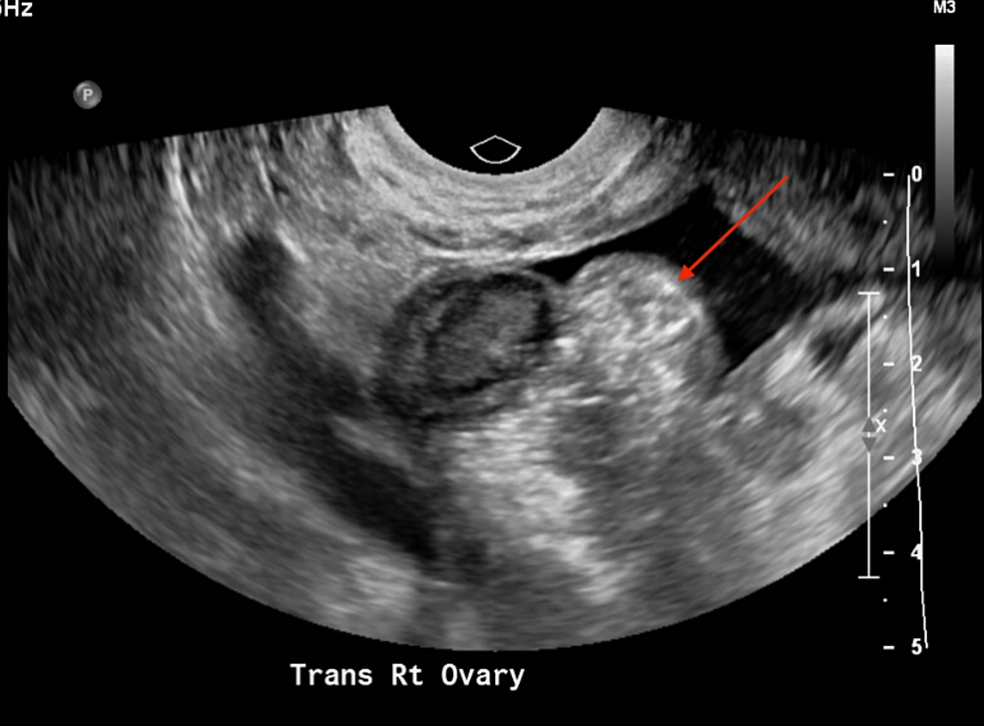
Transvaginal pelvic ultrasound showing right ovary with adjacent heterogeneous soft tissue structure.

CT of the abdomen and pelvis revealed large ascites, presence of fluid between the layers of the mesentery, increased soft tissue density with an 8 mm focal calcification and distortion in the omentum, mild prominence, heterogeneous enhancement of the posterior body of the myometrium, and prominent endometrial complex measuring 9 mm in thickness (Figures 1, 2). The study was limited due to extensive ascitic fluid. For symptomatic ascites, the patient was admitted to medicine for further evaluation. A transvaginal pelvic ultrasound revealed a suspected heterogeneous soft tissue structure in the right adnexal region measuring 3.3 × 1.6 × 2.0 cm (Figure 3) and central endometrial echogenicity. Abdominal ultrasound revealed a coarse hepatic echotexture, mild right hydronephrosis, and ascites with internal echoes.

Serum tumor markers revealed cancer antigen 125 (CA-125) elevated at 214 U/mL, and cancer antigen 19-9 (CA 19-9) was elevated at 53 U/mL. The patient underwent an ultrasound-guided diagnostic and therapeutic paracentesis with the removal of 8.8 L of serosanguinous fluid. Calculated serum ascites albumin gradient (SAAG) was 0, and ascitic fluid and serum lactate dehydrogenase (LDH) ratio was 3.2, suggesting infectious or malignant etiology. Cytologic analysis of ascitic fluid showed multiple clusters of adenocarcinoma cells, favoring Müllerian origin. A preliminary diagnosis of cancer of Müllerian origin was made and the patient was transferred to a tertiary center for an advanced level of care. At the tertiary center, she underwent a repeat CT of the abdomen and pelvis, which revealed moderate ascites, mild peritoneal thickening, and scattered peritoneal calcifications. However, adnexa and endometrium were normal. Again, therapeutic and diagnostic paracentesis was performed with the removal of 350 mL of straw-colored ascitic fluid. Pathology and immunohistochemical studies revealed adenocarcinoma tumor cells that were focally positive for estrogen receptors. Findings were favorable for Müllerian origin and primary peritoneal carcinomatosis. In the absence of an ovarian mass, as outlined in the GOC criteria, a final diagnosis of primary peritoneal carcinomatosis of Müllerian origin was made. Upon symptomatic improvement, the patient was discharged from the hospital with follow-up appointments with hematology/oncology for chemotherapy and continuation of care. Unfortunately, the patient was lost to follow-up with no further electronic records available.

## Discussion

PPC is a rare tumor that arises from the peritoneal lining and mostly involves the omentum [2]. The exact pathogenesis is unknown but is thought to be related to mutations in *TP53*, *KRAS*, *EPHB1*, *BRCA2*, or *BCOR* genes [3]. Women are typically affected more than men, especially those with a family history of ovarian and breast cancer, remarkably with *BRCA1 *and *BRCA2* mutations being associated with an increased lifetime risk of developing PPC [4]. Most patients with an eventual diagnosis of PPC are often at an advanced stage of the disease at diagnosis. In a study by Dalrymple et al., patients with disseminated peritoneal serous papillary carcinoma at the time of diagnosis were clinically and prognostically equivalent to stage III or IV ovarian carcinoma [4]. This highlights the aggressive nature of this malignancy warranting an expeditious diagnosis and management. Histologically, the malignant cells seen in PPC are very similar to ovarian cancer cells. It has been demonstrated that the pelvic peritoneum has the potential to differentiate into Müllerian-type epithelium [5], leading to a diagnostic challenge. Hence, assessment is performed meticulously and other potential primary sites of malignancy, such as the ovaries, gastrointestinal tract, and pancreas, are examined carefully. In our patient, ovarian cancer was initially suspected, but that was later rejected after the removal of a large amount of ascitic fluid showing normal adnexal structures on repeat imaging study.

General presenting symptoms include abdominal pain and ascites. A study analyzing patients from 1976 to 1988 showed that abdominal pain was seen in 68% of patients, with 52% of patients presenting with ascites [6]. Our patient had extensive ascites on presentation and underwent initial paracentesis with the removal of 8.8 L of ascitic fluid with a subsequent paracentesis removing 350 mL. Diagnosis is primarily made using histopathology. Due to cytologic variation, many variants of PPC are seen, including clear-cell PPC, mucinous PPC, endometroid PPC, and a mixed Müllerian mesodermal variant [2]. Tumor markers are also a part of the diagnostic workup including CA-125 which is often elevated creating a diagnostic challenge keeping ovarian cancer as part of the differential.

Some studies have described the differentiating points between PPC and ovarian carcinoma. A study by Barda et al. concluded that a higher rate of abdominal distension, ascites volume, and malignant cells were found in ascitic fluid in PPC compared to ovarian cancer [7]. A comparative study including 20 cases inferred that the patients with ovarian cancer were younger, had a lower baseline CA-125 level, exhibited less severe omental involvement, and had a better response to adjuvant chemotherapy [8]. Treatment of PPC is similar to that of ovarian cancer. Cytoreductive surgery, tumor debulking, chemotherapy, and, rarely, radiotherapy are the modalities of choice. Prognosis depends on the positivity of tumor markers, primary debulking surgery, stage at presentation, and functional status. A retrospective, clinicopathological study reported a median survival period of 23.5 months [9].

Our patient had ascitic fluid findings compatible with PPC, with tumor cells favoring Müllerian origin. Although initially at our hospital an ovarian lesion was suspected, imaging studies were limited due to extensive ascitic fluid. Further workup and imaging studies performed after repeat paracentesis were consistent with PPC without any evidence of an ovarian mass. Unfortunately, no further information regarding management is available for our patient as she was lost to follow-up.

## Conclusions

PPC is a rare and aggressive tumor with a female predominance that often leads to a diagnostic challenge due to its similarity with ovarian cancer in clinical presentation. Imaging studies and analysis of ascitic fluid are imperative to help differentiate PPC. Therefore, meticulous analysis and interpretation of the extensive workup are necessary to aid in timely diagnosis and expeditious therapeutics.
